# Supplementary material for: iCLAP: an innovative method for integrable co-detection of low-abundance antigens with high-plex immunostaining
Source: Nat Commun. 2026 Feb 24;17:3104. doi: 10.1038/s41467-026-69752-y (PMC13039418; doi:10.1038/s41467-026-69752-y)
Supplement: Supplementary file 1 — Supplementary information [file 41467_2026_69752_MOESM1_ESM.pdf]

iCLAP: a novel method for integrable co-detection of low-abundance antigens with high-plex immunostaining

Fan Wu, Shuyuan Zheng, Yani Chen, Peijia Ye, Moo Joong Kim, Seojin Lee, Geroge Kuo, Shriya Pillan, Ruihan Yuan, Kyu Sang Han, Bofei Yu, Qingfeng Zhu, Sarah M. Shin, Courtney D. Cannon, Gabriele Pierre, Kanako Iwasaki, Cristina Aguayo-Mazzucato, Nicolas Musi, George A. Kuchel, Birgit Schilling, Laura D. Wood, Won Jin Ho, Robert A. Anders, Denis Wirtz, Pei-Hsun Wu

Supplementary Information

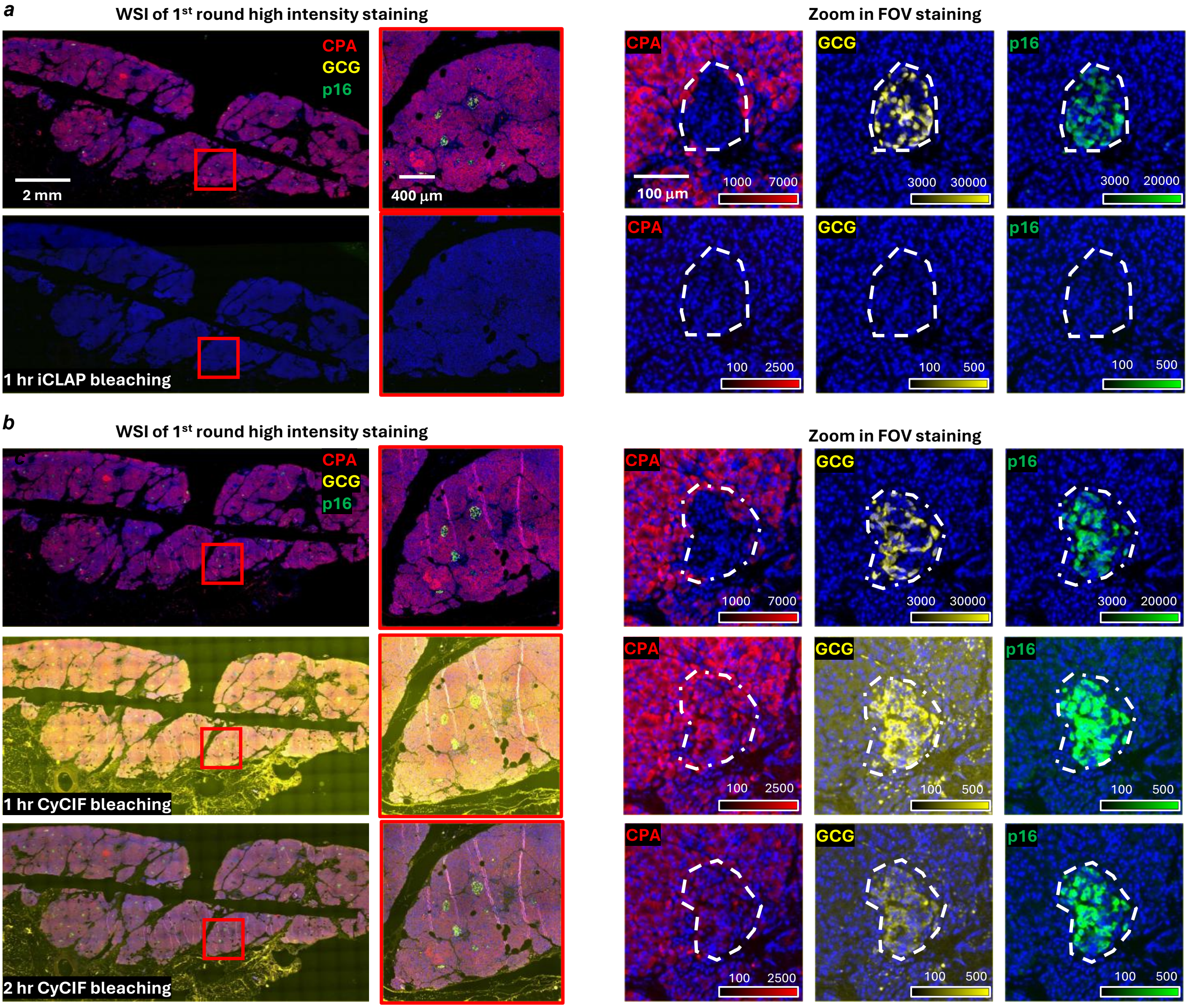

Supplementary Figure 1. Comparison of iCLAP bleaching methods vs CyCIF bleaching method. a. TSA-based high-intensity staining of CPA (red), GCG (yellow), and p16 (green) before and after one-hour iCLAP bleaching at various magnifications. **b.** TSA-based high-intensity staining of CPA (red), GCG (yellow), and p16 (green) before and after one or two hours of CyCIF bleaching at various magnifications. Due to substantial DAPI signal reduction after iCLAP bleaching and CyCIF bleaching, the DAPI channel displayed represents the aligned DAPI signal from the first-round staining, used to maintain visualization of tissue architecture. The results demonstrate that the iCLAP bleaching method effectively reduce TSA-based high-intensity staining to background levels, while the same duration or even longer periods of CyCIF bleaching still left noticeable residual fluorescence signals. All images are representative of at least three independent staining and bleaching experiments performed on independent tissue sections, with similar results observed. The pseudocolor intensity scale bar shown at the bottom right indicates fluorescence intensity values (arbitrary units).

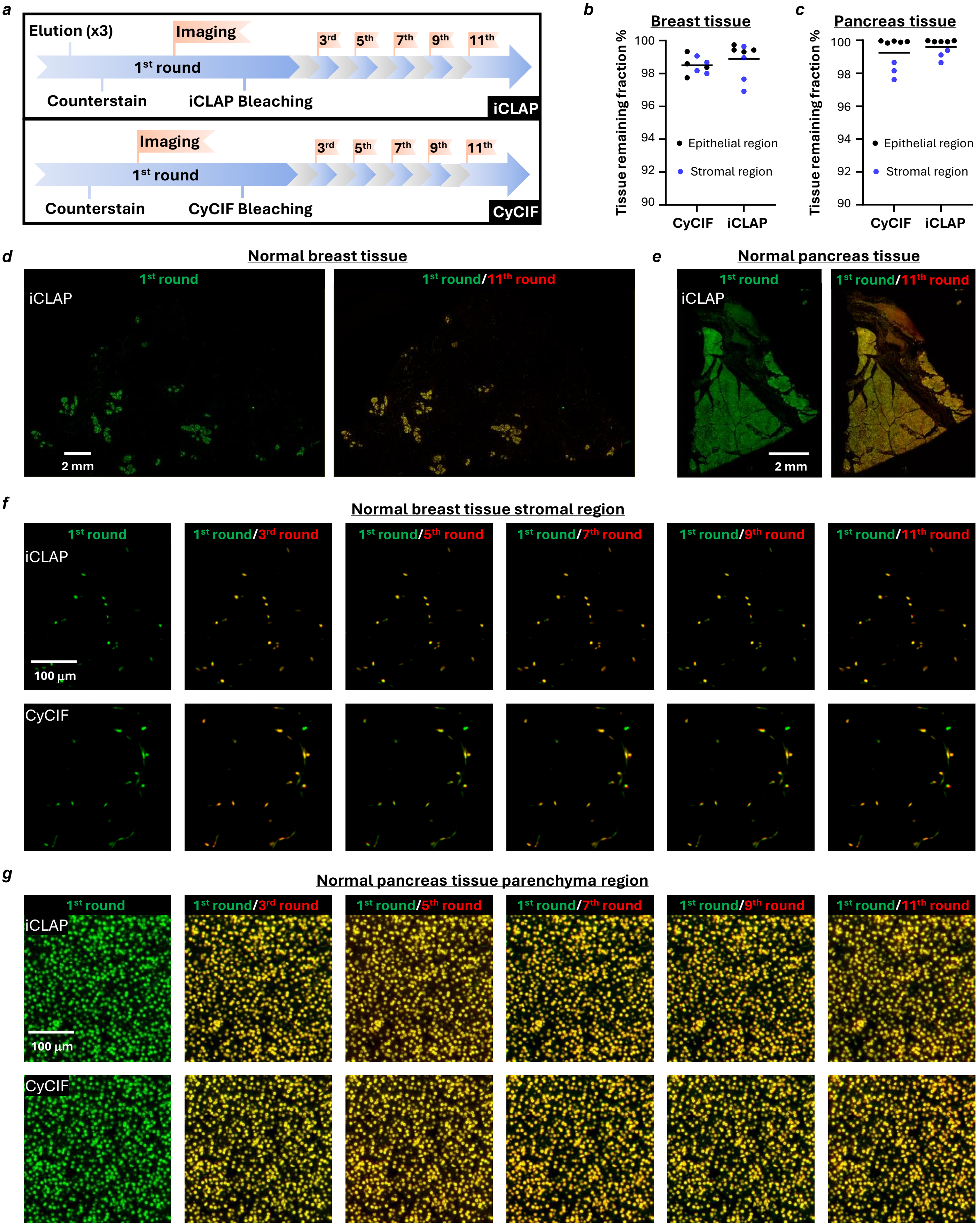

a
Elution (x3)
Imaging
3rd
5th
7th
9th
11th
1st round
Counterstain
iCLAP Bleaching
iCLAP
Imaging
1st round
Counterstain
CyCIF Bleaching
CyCIF
b
Breast tissue
Tissue remaining fraction %
Epithelial region
Stromal region
CyCIF
iCLAP
c
Pancreas tissue
d
Normal breast tissue
1st round
1st round/11th round
2 mm
e
Normal pancreas tissue
f
Normal breast tissue stromal region
1st round/3rd round
1st round/5th round
1st round/7th round
1st round/9th round
100 μm
g
Normal pancreas tissue parenchyma region

Supplementary Figure 2. Comparison of tissue retention across multiple rounds of staining using iCLAP and CyCIF workflows. a. Schematic overview of the tissue processing workflows in iCLAP and CyCIF across 11 staining cycles. In the iCLAP workflow (top), each staining round begins with three sequential antibody elution steps, followed by counterstaining and iCLAP bleaching. Imaging is performed after every two staining-bleaching cycles (i.e., 1st, 3rd, 5th, etc.), up to the 11th round. In contrast, the CyCIF workflow (bottom) involves direct counterstaining and CyCIF bleaching steps without antibody elution steps, with imaging also conducted at every odd-numbered round. **b-c.** Quantification of tissue retention across staining rounds in normal breast (**b**) and pancreas (**c**) tissues. Tissue area was measured separately in epithelial and stromal regions across 11 rounds, showing similar retention between CyCIF workflow and iCLAP workflow. N = 8. **d–e.** Whole slide images of normal breast (**d**) and pancreas (**e**) tissues showing preservation of DAPI-stained nuclei from round 1 (green) and round 11 (red), with minimal tissue dropout across both iCLAP and CyCIF workflows. **f–g.** High-magnification images from normal breast stromal regions (**f**) and pancreatic parenchyma (**g**) confirm consistent nuclear density and indicate that tissue retention remains comparable between CyCIF and iCLAP workflows throughout all staining rounds.

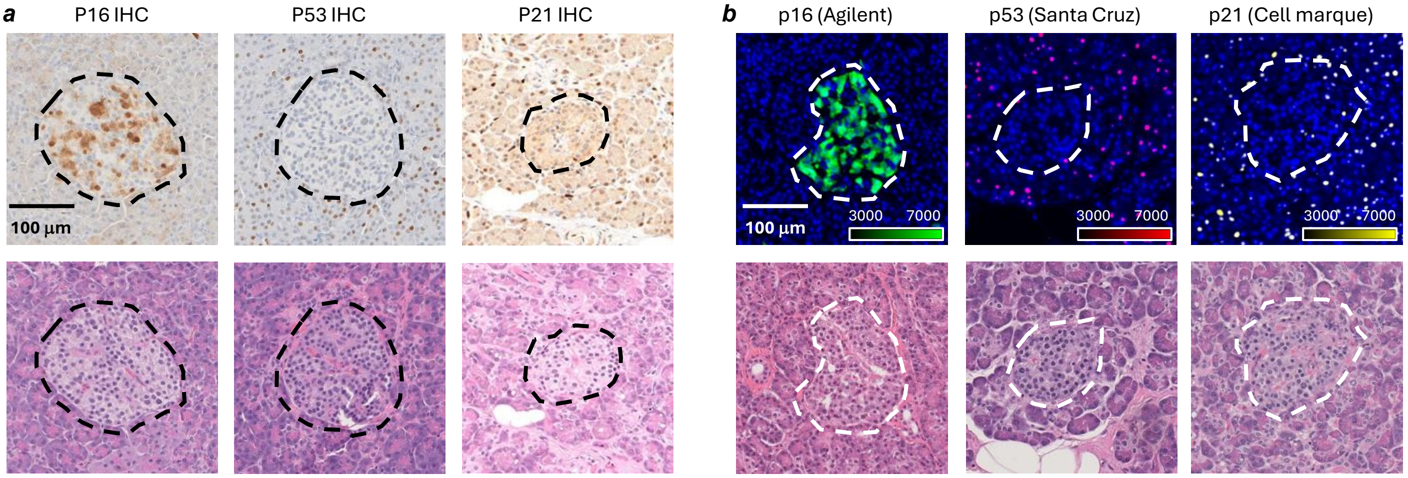

Supplementary Figure 3. TSA staining validation. a. Immunohistochemical (IHC) staining of p16, p53, and p21 performed by clinical laboratory in healthy human pancreas tissue samples showed staining patterns consistent with iCLAP. P16 positive clusters predominantly located in islets of Langerhans regions; p53 positive nucleus mainly located in the acini regions, and p21-positive nuclei predominantly in acinar regions. **b.** TSA detection of p16, p53, and p21 using antibodies from different vendors showed similar pattern, further confirm the staining pattern observed. For each IHC or IF image, adjacent H&E-stained image was shown below. Representative images are shown from at least three independent tissue donors with consistent staining patterns. The pseudocolor intensity scale bar shown at the bottom right indicates fluorescence intensity values (arbitrary units).

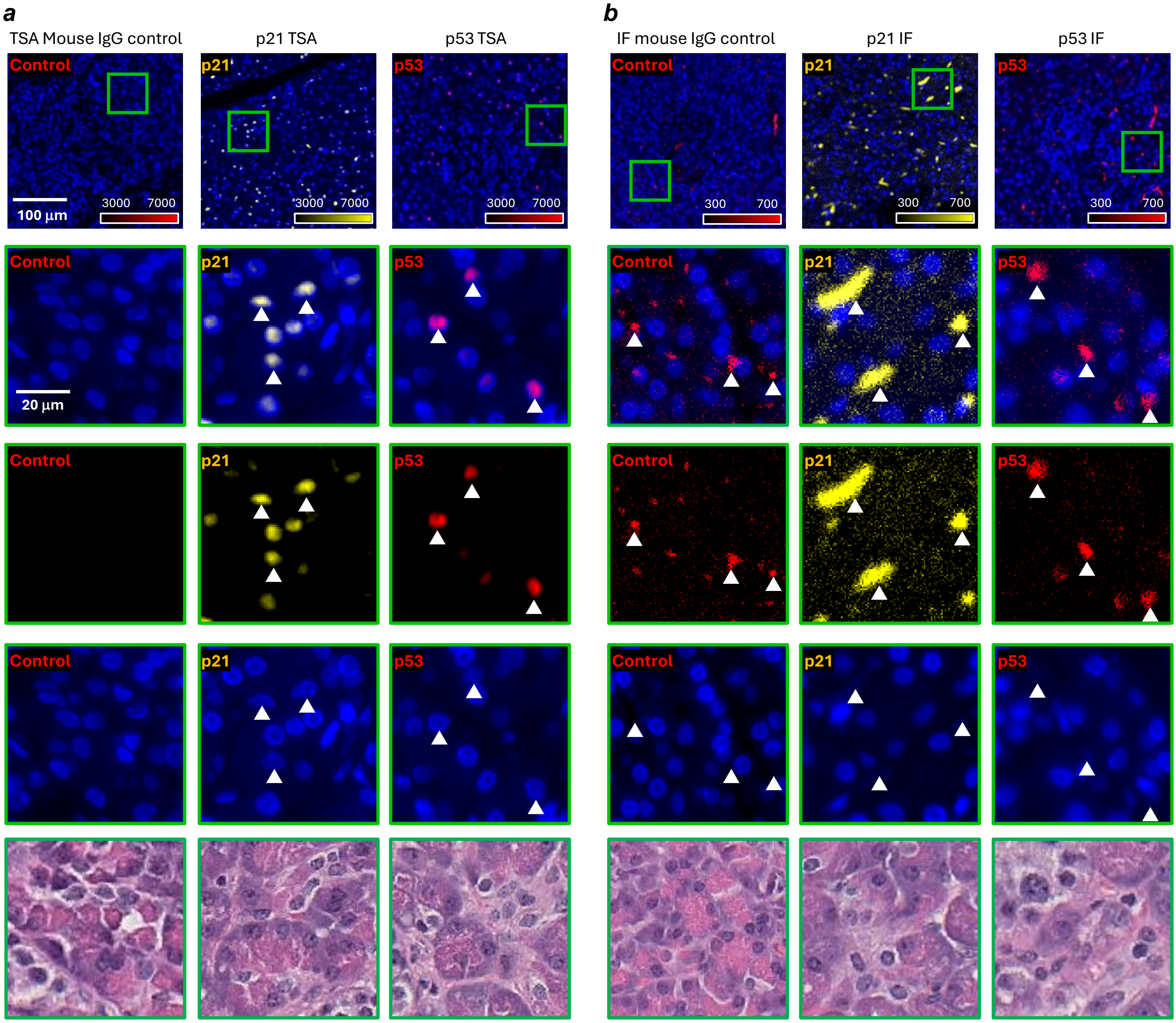

Supplementary Figure 4. Comparison of p53 and p21 staining using TSA and immunofluorescence (IF). a. TSA based mouse IgG control, p53 and p21 staining reveals strong nuclear localization of both p53 and p21 compared to IgG control slides, indicating specific staining of these proteins within the nucleus. **b.** In comparison, conventional IF staining for p53 and p21 shows minimal nuclear localization and predominantly cytoplasmic or diffuse background signals, suggesting weak sensitivity for low-abundance nuclear targets under standard IF conditions. For each selected high-magnification regions, adjacent H&E-stained images are shown below. Representative images are shown from at least three independent tissue donors with consistent staining patterns. The pseudocolor intensity scale bar shown at the bottom right indicates fluorescence intensity values (arbitrary units).

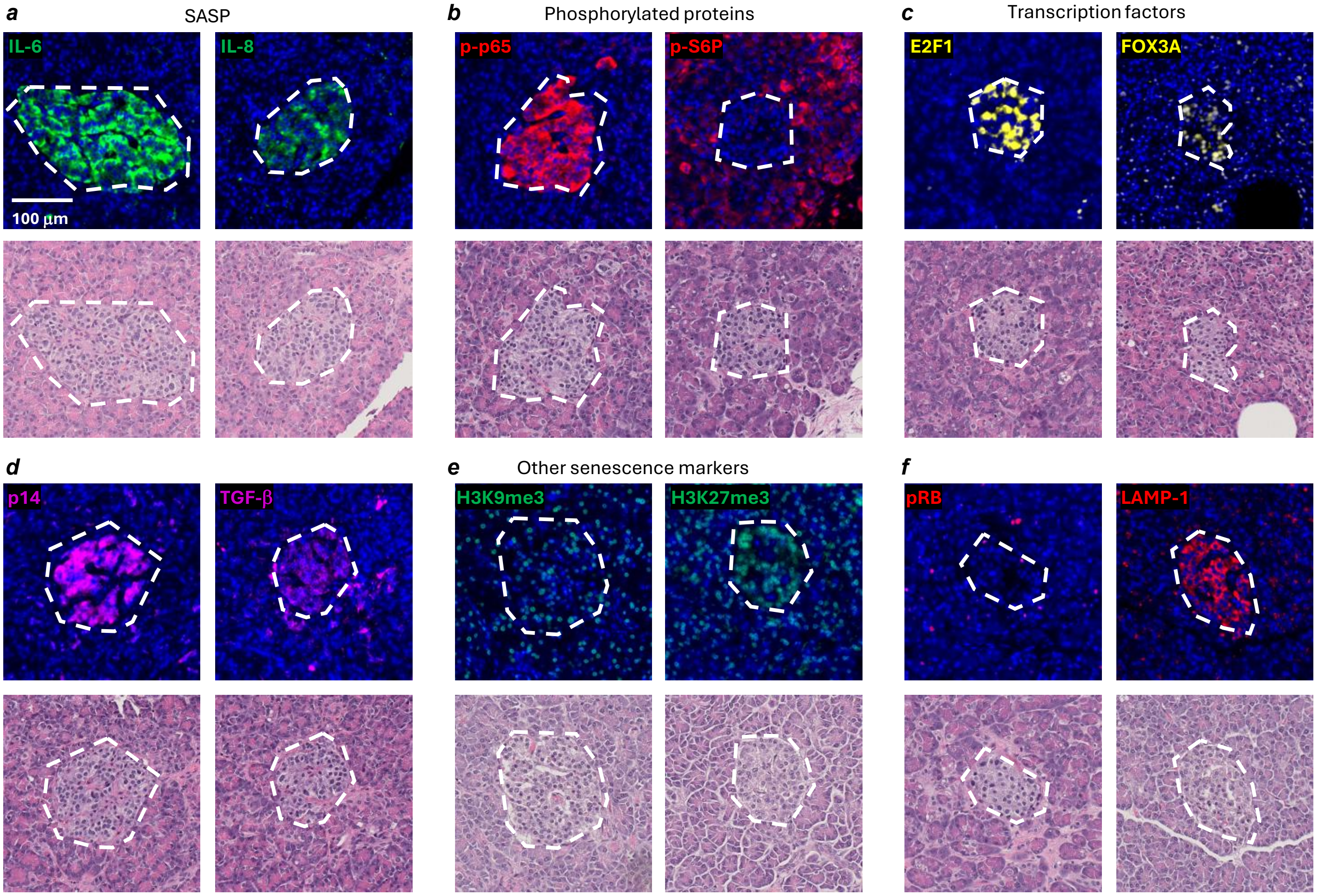

Supplementary Figure 5. TSA-based staining of other low abundance senescence related proteins. a. TSA-based senescence-associated secretory phenotype (SASP) IL-6 and IL-8 staining of a human pancreas slide. **b.** TSA-based senescence-associated phosphorylated proteins p-p65 and p-S6P staining of a human pancreas slide. **c.** TSA-based senescence-associated transcription factor E2F1 and FOXO3A staining of a human pancreas slide. **d-f.** TSA-based senescence-associated markers p14, TGF-beta, H3K9me3, H3K27me3, pRB and LAMP-1 staining of a human pancreas slide. For each marker, adjacent H&E-stained images are shown below. Representative images are shown from at least three independent tissue sections with consistent staining patterns.

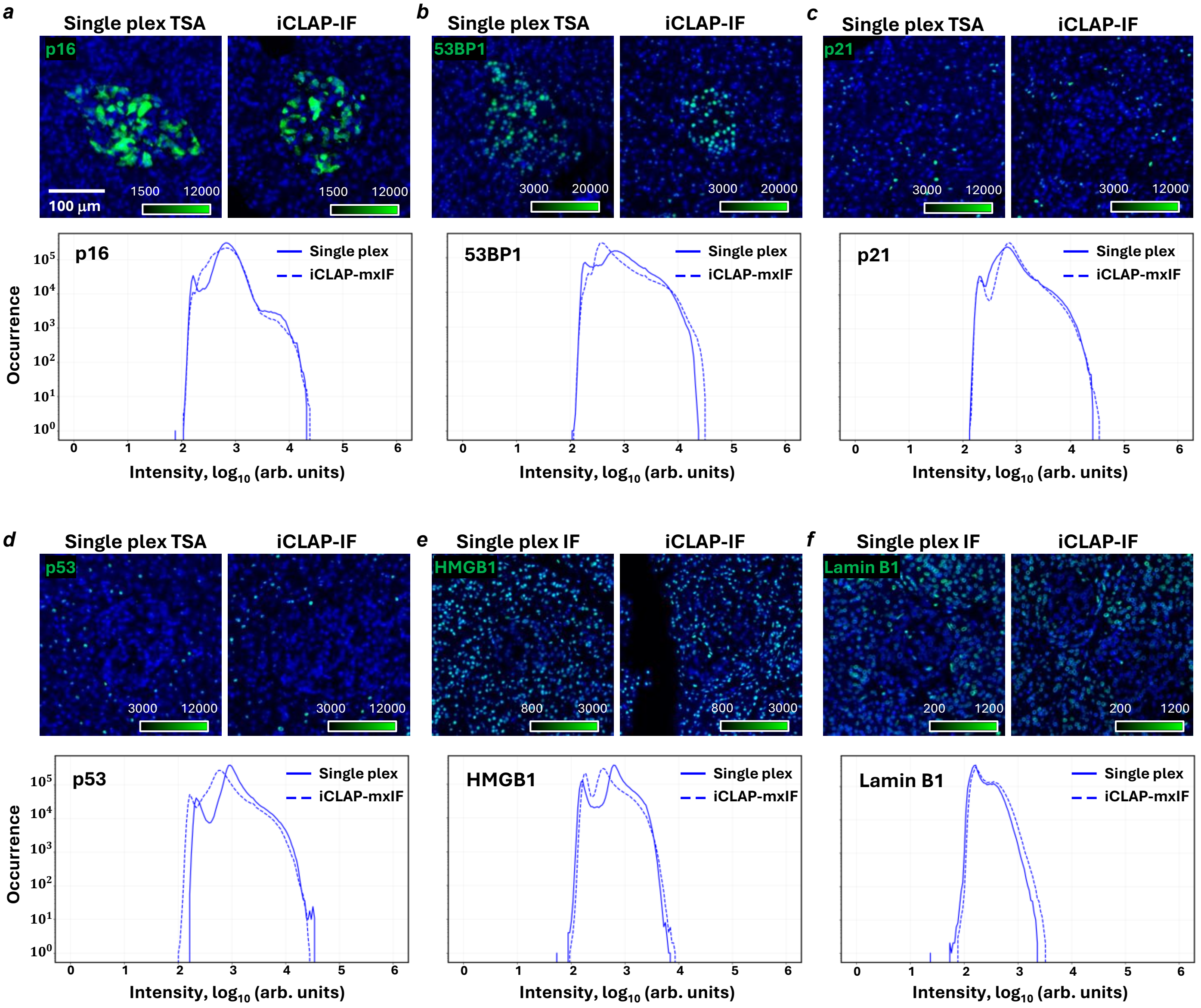

Supplementary Figure 6. Multiplex iCLAP-IF preserves signal intensity compared to single-plex staining. a–f. Representative images (top) show staining for P16 (**a**), 53BP1 (**b**), P21 (**c**), P53 (**d**), HMGB1 (**e**), and Lamin B1 (**f**) using conventional single-plex TSA/IF and multiplex iCLAP-IF. Quantitative fluorescence intensities (bottom, log10 scale) show that iCLAP-IF staining closely matches the intensity distributions of single-plex staining, confirming minimal loss of sensitivity with six-plex detection. The pseudocolor intensity scale bar shown at the bottom right indicates fluorescence intensity values (arbitrary units).

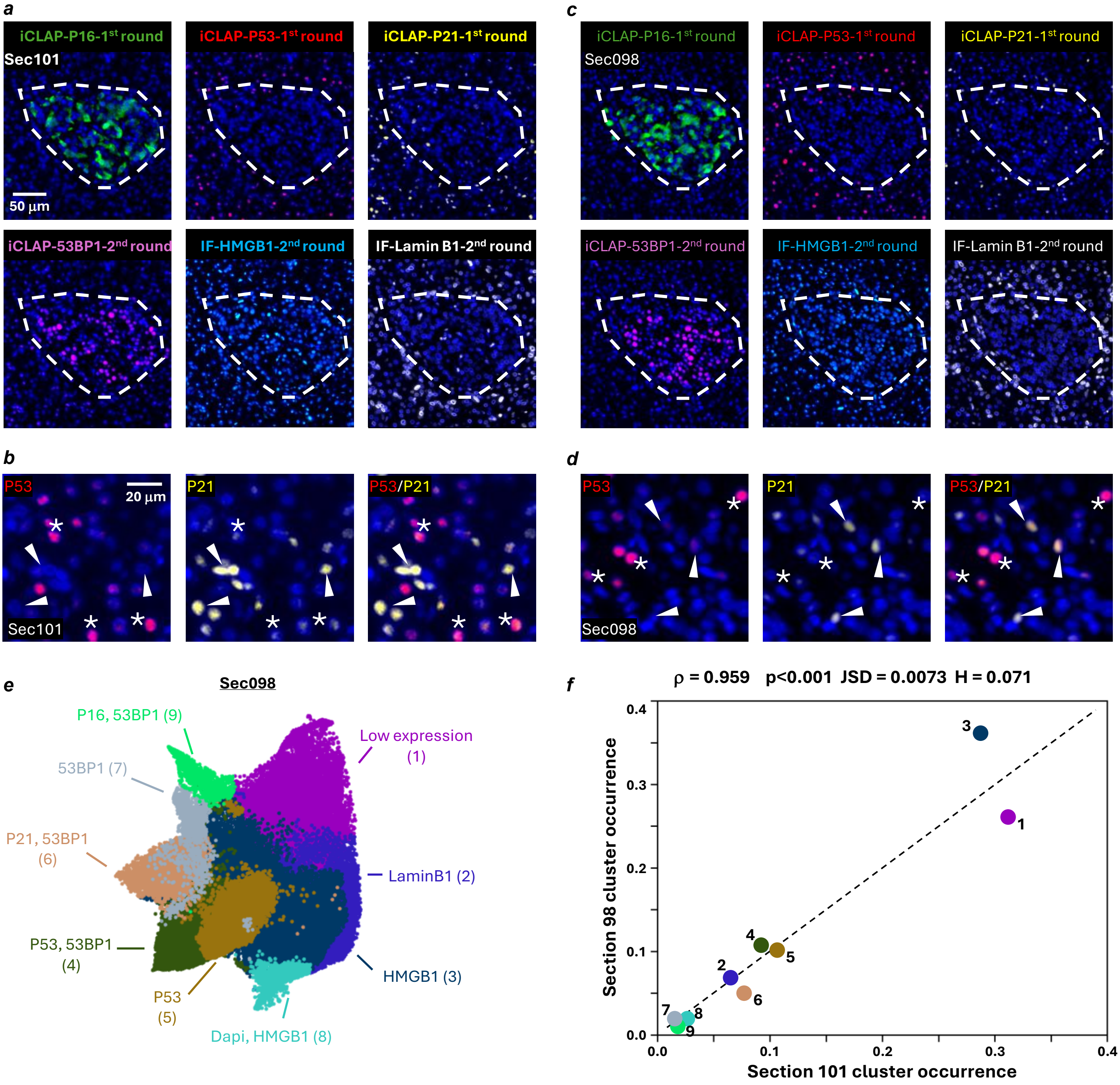

Supplementary Figure 7: Reproducibility of staining pattern, UMAP embedding, and cluster occurrence across adjacent sections. a. Single-channel images of six senescence markers detected by iCLAP-IF, corresponding to Figure 3c. **b.** High-magnification images from the boxed acinar region in panel (**a**), showing single-channel P53 (red) and P21 (yellow) expression. These images show individual nuclei that distinctly express either P53 (asterisks) or P21 (arrowheads), but not both. The merged channel confirms limited colocalization of these two markers, indicating separate senescence phenotypes in acinar cells. **c-d.** Adjacent iCLAP-IF-stained section of the same islet region showing consistent marker expression patterns. **e.** UMAP analysis of senescence marker cluster distributions in section 098, generated using identical clustering parameters as section 101. The qualitative similarity between the two sections demonstrates stable and reproducible clustering of marker expression across adjacent sections. **f.** Cluster occurrence is highly concordant across slides (Pearson (two-tailed) r=0.959, two-tailed $p=4.3\times10^{-5}$; 95% CI 0.813–0.992), with near-identical distributions confirmed by low Jensen–Shannon divergence (0.0073) and Hellinger distance (0.071).

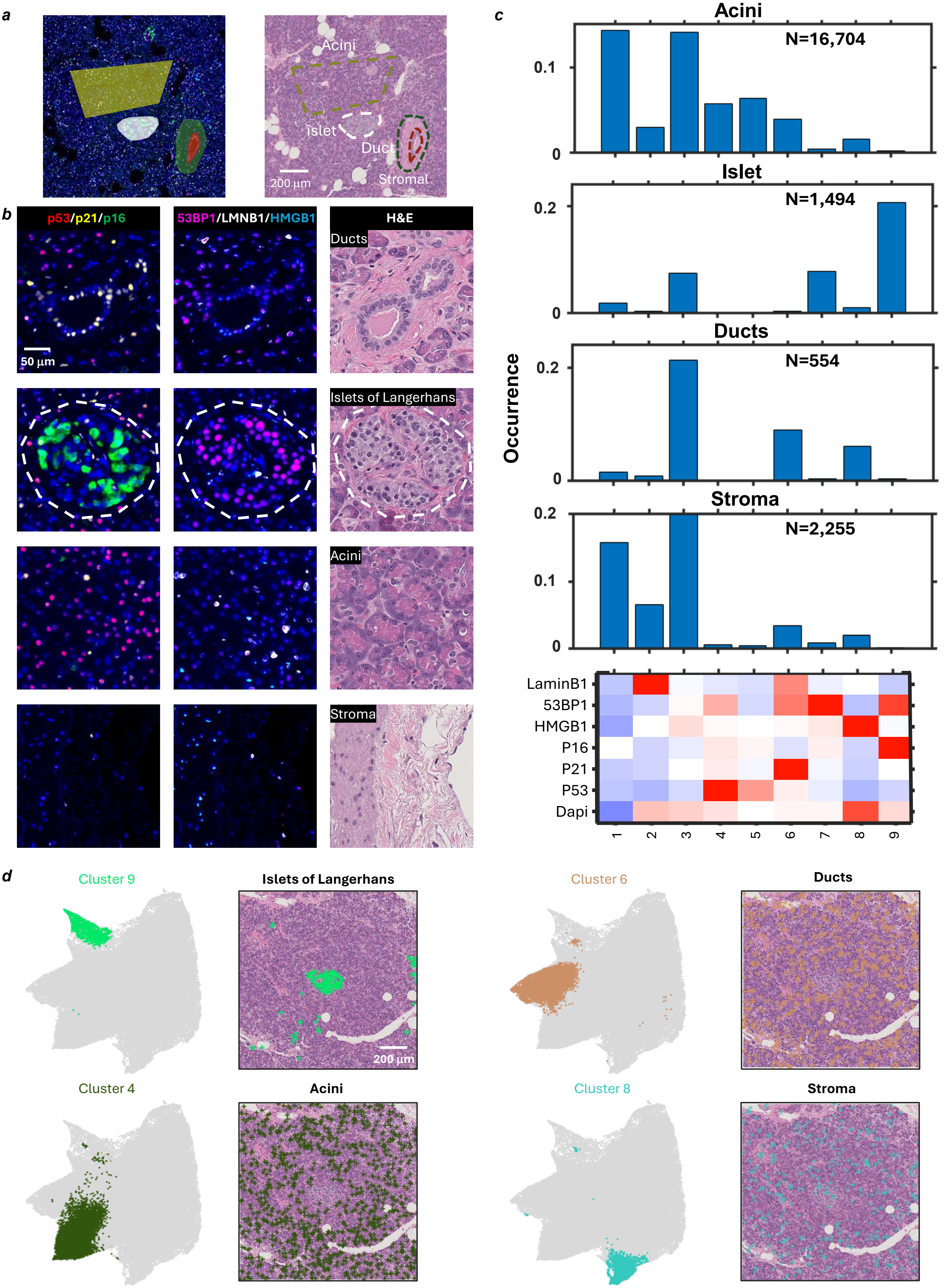

a
Acini
islet
Duct
Stromal
200 µm
b
p53/p21/p16
53BP1/LMNB1/HMGB1
H&E
Ducts
50 µm
Islets of Langerhans
Acini
Stroma
c
Acini
N=16,704
Islet
N=1,494
Ducts
N=554
Stroma
N=2,255
Occurrence
LaminB1
53BP1
HMGB1
P16
P21
P53
Dapi
1 2 3 4 5 6 7 8 9
d
Cluster 9
Islets of Langerhans
200 µm
Cluster 6
Ducts
Cluster 4
Acini
Cluster 8
Stroma

Supplementary Figure 8. iCLAP-IF 6 plex panel enables senescence marker detection in various FFPE tissue types. a. Illustration of using adjacent H&E images to guide annotation of the acinar, islets, stromal, ductal region in 6 plex senescence markers image. **b**. Immunofluorescence staining of senescence markers (P53, P21, P16, 53BP1) and nuclear markers (LaminB1, HMGB1) across different pancreatic compartments, with matched H&E images for structural reference. **c**. Quantitative analysis of cell distribution across pancreatic compartments (acini, islets, ducts, and stroma) based on senescence expression clusters, accompanied by a heatmap depicting marker expression patterns within each cluster. The results highlights clusters 9, 6, 4, and 8 are highly associated with the islets of Langerhans, ducts, acini, and stroma, respectively. **d**. Locations of senescence expression clusters 9, 6, 4 and 8 in UMAP space and corresponding adjacent H&E images.

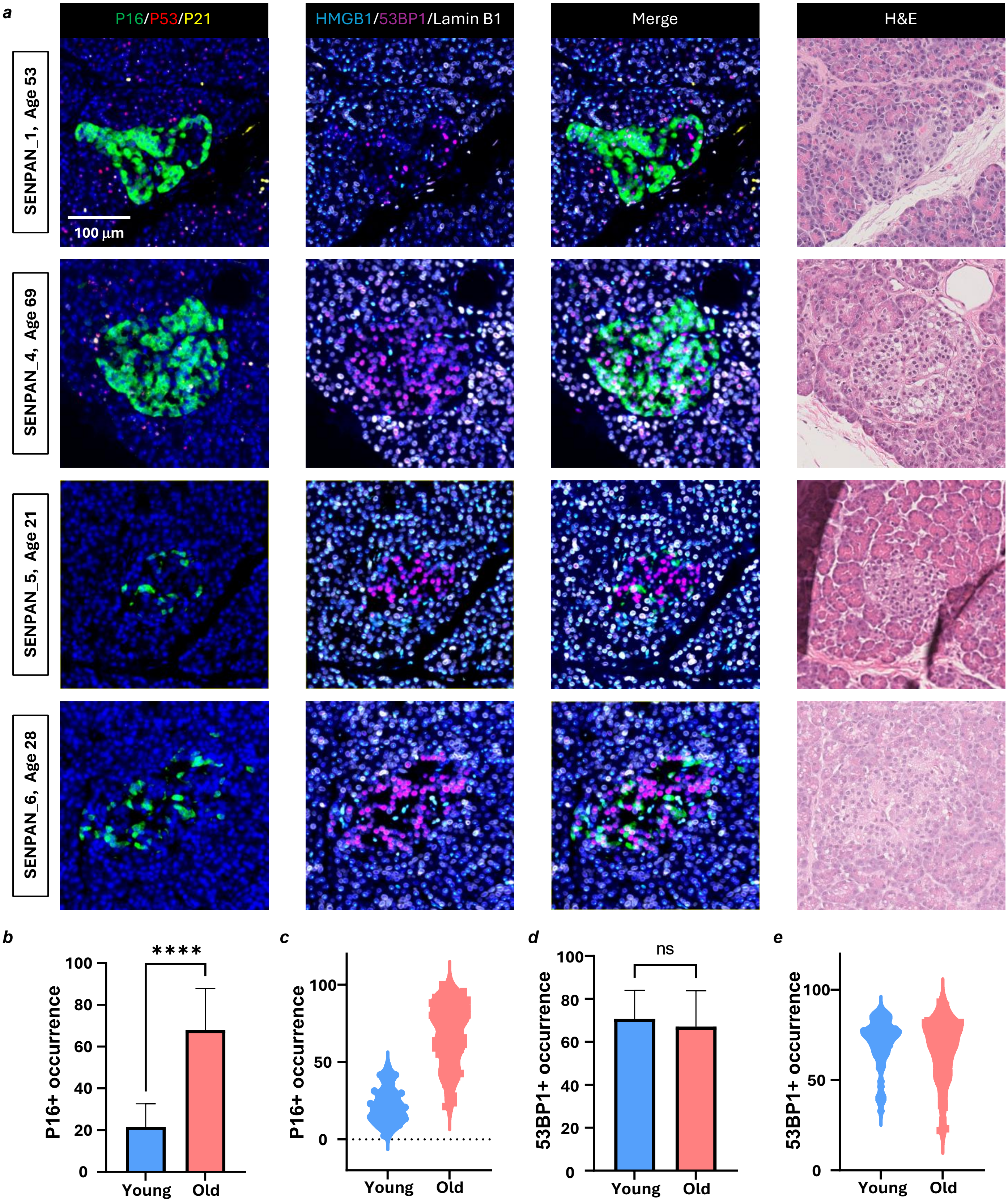

a
P16/P53/P21
HMGB1/53BP1/Lamin B1
Merge
H&E
SENPAN_1, Age 53
100 µm
SENPAN_4, Age 69
SENPAN_5, Age 21
SENPAN_6, Age 28
b
****
P16+ occurrence
0
20
40
60
80
100
Young
Old
c
P16+ occurrence
0
50
100
Young
Old
d
ns
53BP1+ occurrence
0
20
40
60
80
100
Young
Old
e
53BP1+ occurrence
0
50
100
Young
Old

Supplementary Figure 9. Six-plex iCLAP-IF across donors. a. Representative 6 plex senescence iCLAP-IF images from multiple donors. Adjacent H&E-stained images are shown at right. **b-c.** Quantification across 5 donors(young n = 2, old n = 3; 30 islets per donor) shows a significant increase in P16+ occurrence (fraction of marker-positive cells per islet) in older versus younger donors. **d-e.** 53BP1+ occurrence in islets does not differ significantly between age groups. Two-sided unpaired t-test (Welch's) was applied to assess differences in islet-level P16+ and 53BP1+ occurrence between young and old donors. Data are presented as mean ± standard deviation (SD), and bars show mean ± SD and violins display all islet-level values (occurrence = fraction of P16+ cell per islet). Results were considered significant at $P < 0.05$ (*), $P < 0.01$ (**), and $P < 0.001$ (***), $P < 0.0001$ (****). "ns" indicates no significant difference.

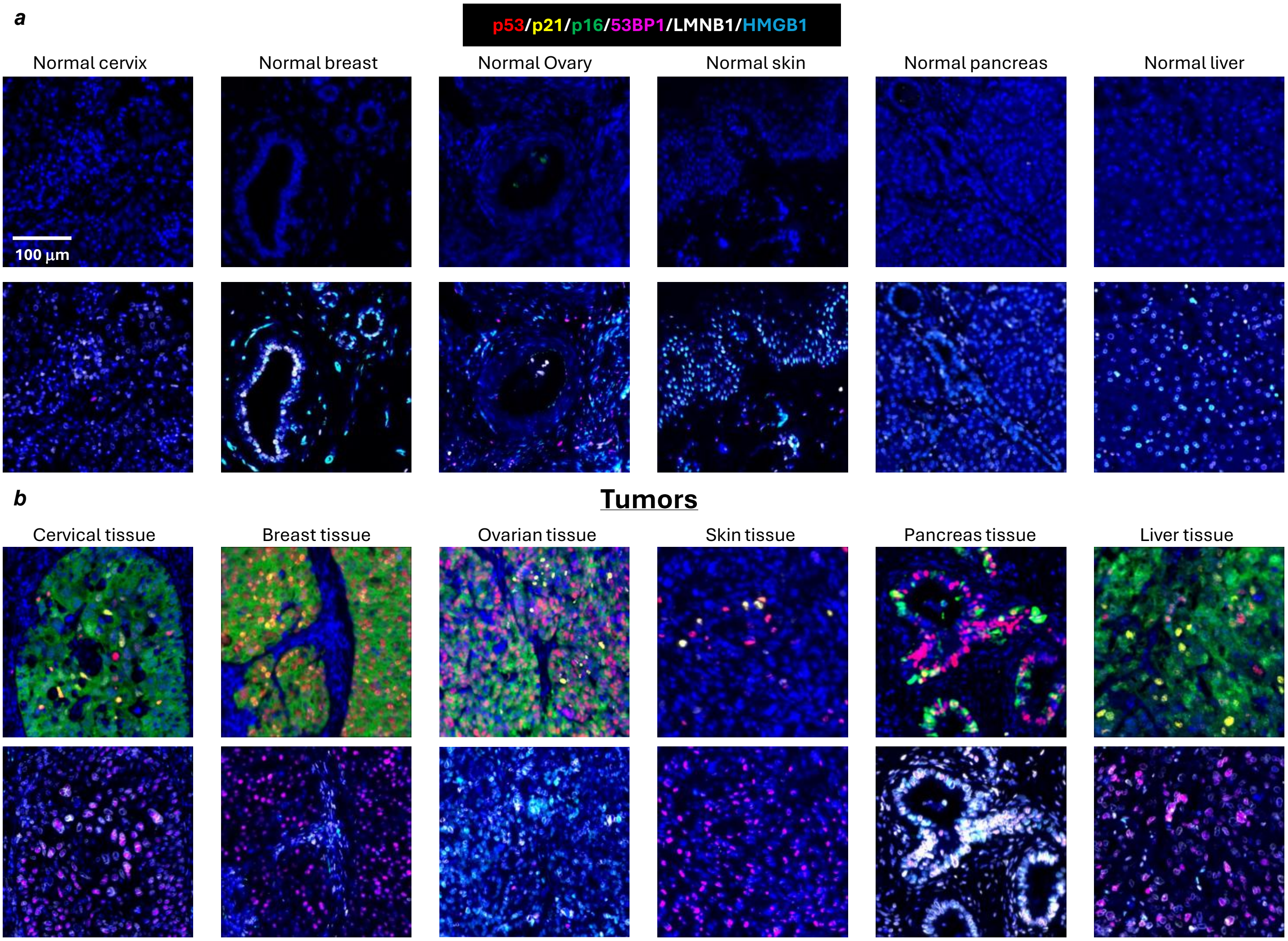

Supplementary Figure 10. Six-plex iCLAP-IF across normal and tumor tissues. a. 6 plex senescence markers panel staining in normal cervical, breast, ovarian, skin, pancreas, and liver tissue. **b.** 6 plex senescence markers panel staining in tumor cervical, breast, ovarian, skin, pancreas, and liver tissue. Representative images are shown from at least 10 independent TMA cores per tissue type.

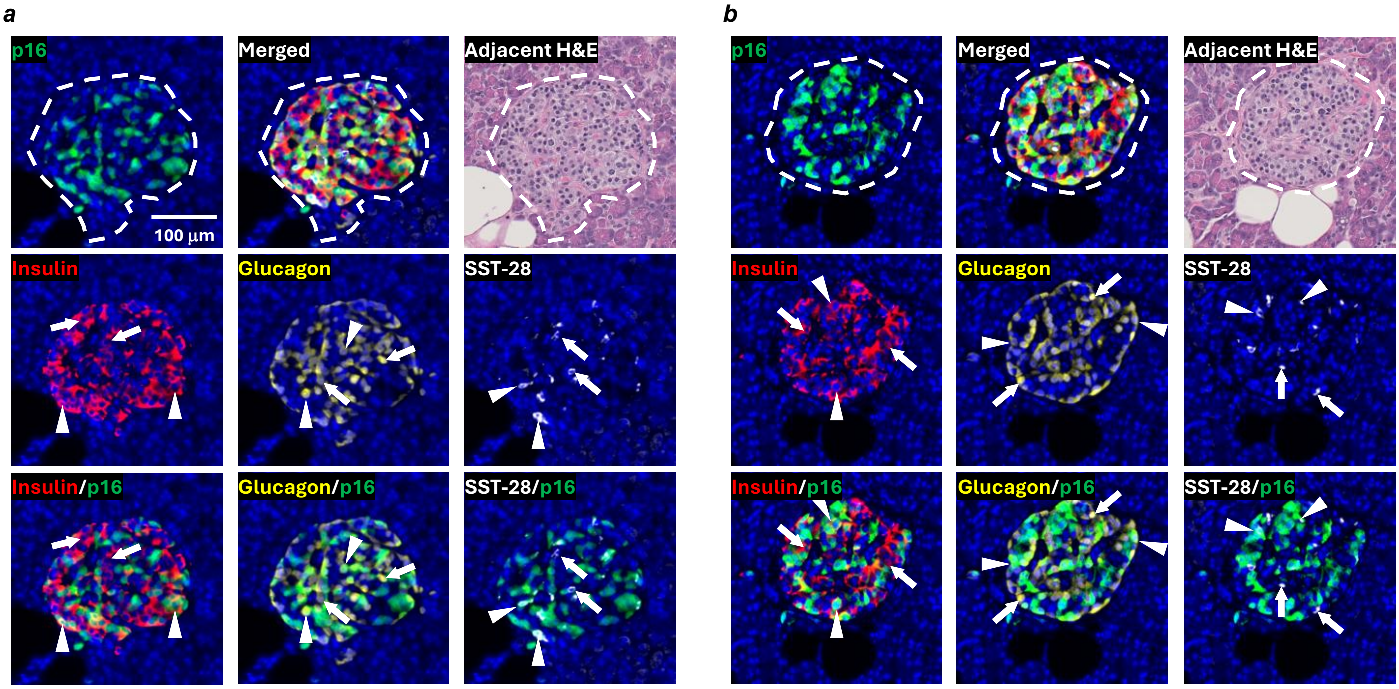

Supplementary Figure 11. Heterogeneous P16 expression across pancreatic islet cell subtypes. a-b. Multiplexed iCLAP-CyCIF images of pancreatic islets shows that all three endocrine cell subtypes—Insulin+ (red, beta cells), Glucagon+ (yellow, alpha cells), and SST-28+ (white, delta cells)—can exhibit both P16+ (green) and P16− signatures. Arrowheads and arrows indicate examples of P16+ and P16− endocrine cells within different islets subtypes. Adjacent H&E staining provides tissue morphology for reference.

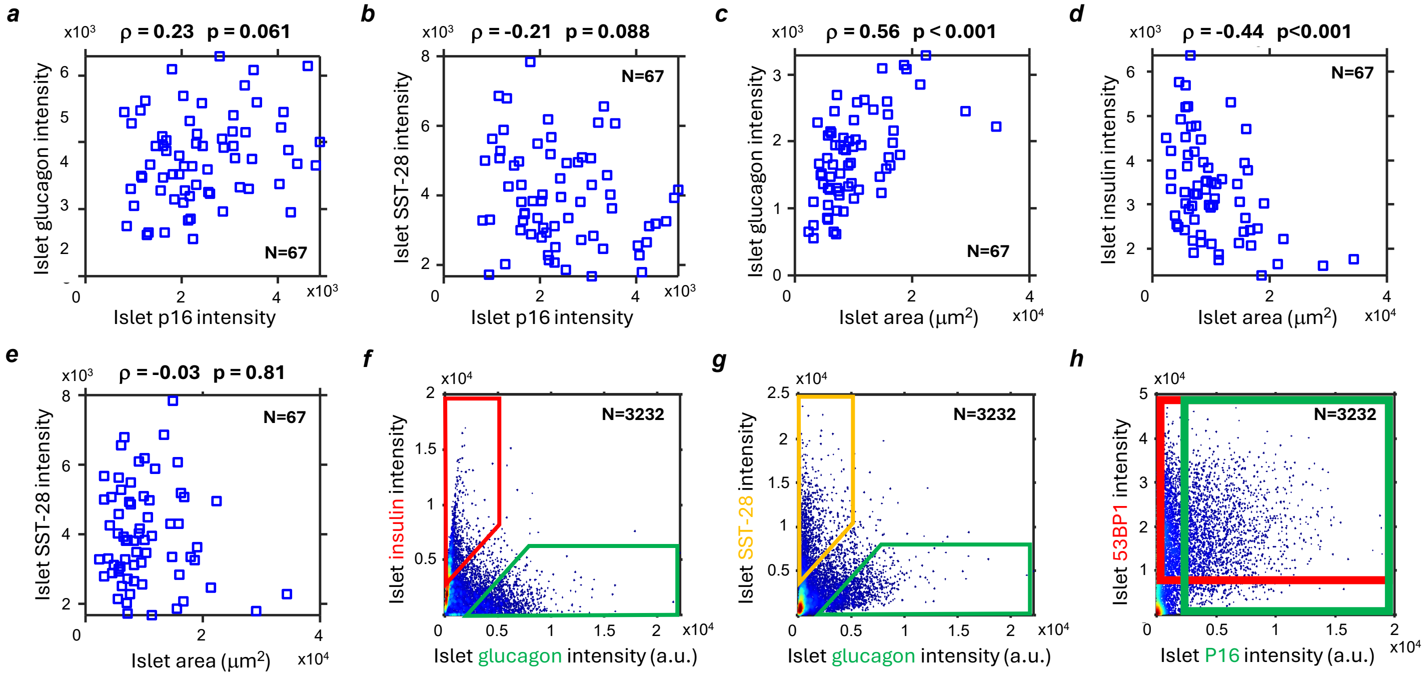

Supplementary Figure 12. Islet level correlation analysis of senescence, pancreatic functional markers, and islet morphology. a-b. Scatter plots show Islet-level correlation between p16 and glucagon expression (in arbitrary units) (Pearson correlation coefficient (two-tailed), ρ = 0.23; p-value = 0.061) and between p16 and SST-28 expression (ρ = -0.21; p-value = 0.088), N=67 islets. **c-e**. The scatter plots show Islet-level correlation between islet area with islet glucagon expression (ρ = 0.56; p-value < 0.001), islet insulin expression (ρ = -0.44; p-value < 0.001), and islet SST-28 expression (ρ = -0.03; p-value = 0.81), respectively, N=67 islets. **f-g.** The scatter plots show islets cells were classified into 3 subtypes: Insulin+ beta cells (red), glucagon+ alpha cells (green), SST-28+ delta cells (yellow), N=3232 cells. **h.** The scatter plots show islets cells were also categorized based on P16 and 53BP1 expression, N=3232 cells.

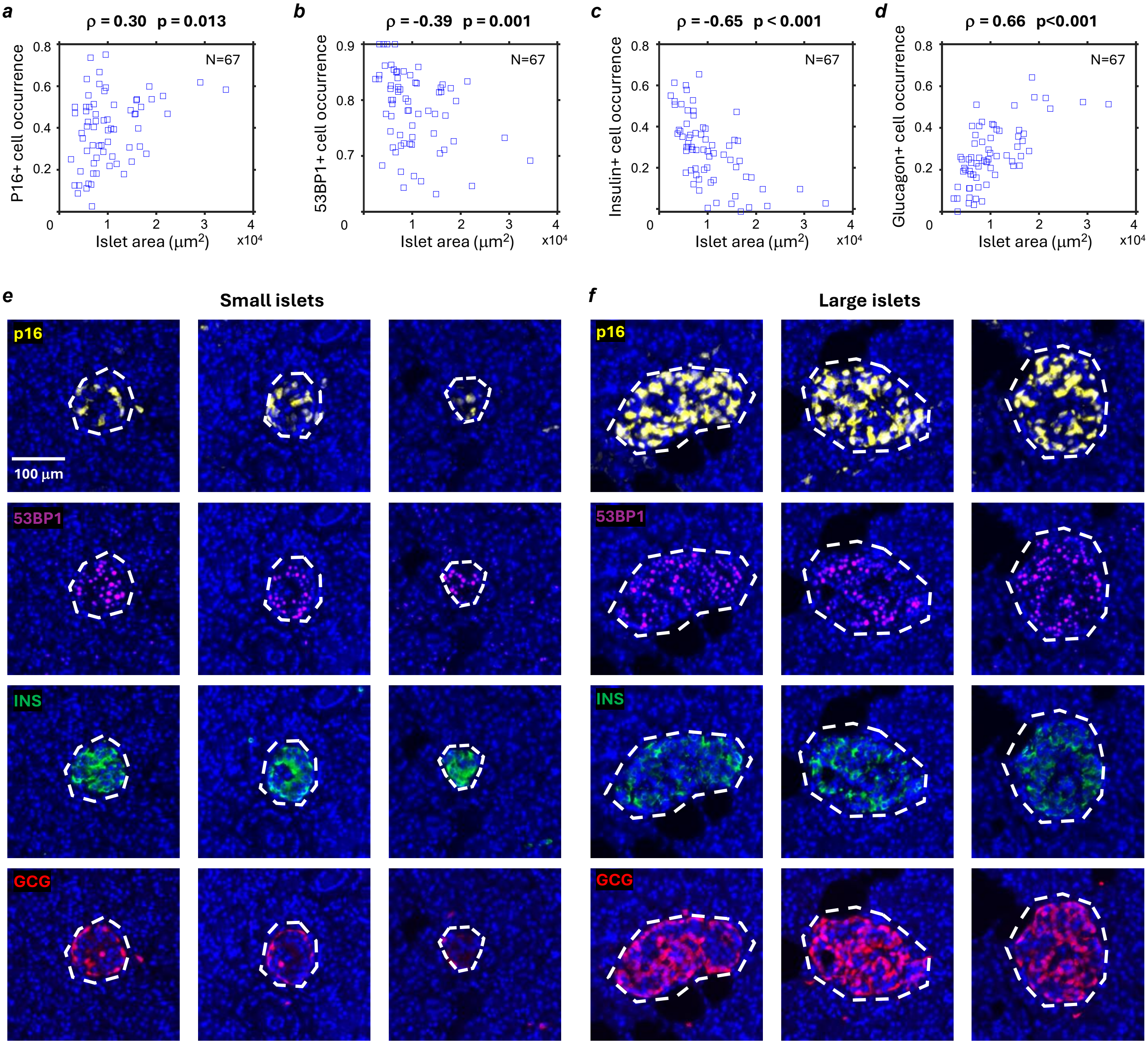

Supplementary Figure 13. Analysis of senescence, pancreatic functional markers, and islet morphology. a-d. The scatter plots show correlations between islet area and occurrence of cells positive for p16 (Pearson correlation coefficient (two-tailed), ρ = 0.30; p-value = 0.013), 53BP1 (ρ = -0.39; p-value = 0.001), INS (ρ = -0.65; p-value < 0.001), and GCG (ρ = 0.66; p-value < 0.001), N=67 islets. **e-f.** Representative iCLAP-CyCIF images showing expression of cellular markers in small (e) and large (f) islets. Larger islets exhibit a higher occurrence of Glucagon (red) and P16 (yellow)-positive cells, while showing a reduction in Insulin (green) and 53BP1 (magenta)-positive cells compared to smaller islets.

a

	All	P16-/53BP1-	P16-/53BP1+	P16+/53BP1-	P16+/53BP1+
**Beta cells (insulin+)**					
Cell Count	1726	173 (10.2%)	857 (49.6%)	17 (1.0%)	679 (39.3%)
Nuclear area (μm^2)	26.6 ± 8.9	19.2 ± 5.3	28.1 ± 8.9	17.6 ± 3.7	26.9 ± 8.8
Insulin Intensity ($\times 10^3$)	2.82 ± 1.73	2.34 ± 1.47	2.72 ± 1.64	3.30 ± 1.75	3.05 ± 1.87
Dapi Intensity ($\times 10^4$)	1.03 ± 0.30	1.27 ± 0.45	0.99 ± 0.25	1.07 ± 0.32	1.00 ± 0.27
P16 Intensity ($\times 10^3$)	2.25 ± 2.50	0.66 ± 0.59	0.70 ± 0.50	3.96 ± 1.49	4.70 ± 2.39
53BP1 intensity ($\times 10^4$)	2.00 ± 0.96	0.19 ± 0.13	2.19 ± 0.78	0.27 ± 0.13	2.20 ± 0.77
**Alpha cells (glucagon+)**					
Cell Count	1292	164(12.7%)	585 (45.3%)	46 (3.6%)	497 (38.5%)
Nuclear area (μm^2)	22.6 ± 6.4	19.0 ± 4.4	23.4 ± 6.6	18.1 ± 3.9	23.3 ± 6.3
Glucagon Intensity ($\times 10^3$)	4.18 ± 2.19	2.96 ± 2.69	4.51 ± 2.07	3.94 ± 3.43	4.21 ± 1.82
Dapi Intensity ($\times 10^4$)	1.25 ± 0.33	1.27 ± 0.37	1.26 ± 0.33	1.07 ± 0.31	1.26 ± 0.32
P16 Intensity ($\times 10^3$)	2.35 ± 2.46	0.66 ± 0.63	0.77 ± 0.56	4.32 ± 1.90	4.58 ± 2.37
53BP1 intensity ($\times 10^4$)	1.18 ± 0.70	0.26 ± 0.17	1.35 ± 0.64	0.38 ± 0.13	1.35 ± 0.63

b

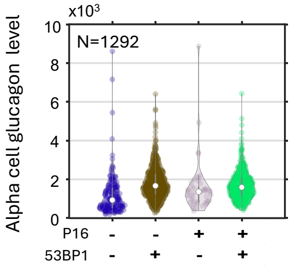

c

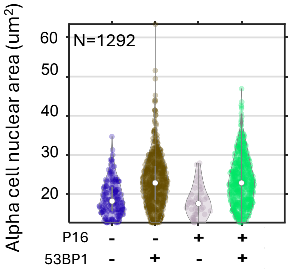

Supplementary Figure 14. Single cell level analysis of P16/53BP1 and functional markers in pancreatic islets. a. Single cell level analysis of 3018 pancreatic islets cells and their P16/53BP1 expression. **b.** Violin plot shows the alpha cell glucagon levels stratified by p16 and 53BP1 expression. **c.** Alpha cell nuclear size distribution across different p16 and 53BP1 expression groups. The white dot indicates the median, and the gray vertical line indicates the interquartile range (25th–75th percentile). Sample sizes are indicated in each panel. Summary statistics, including mean, median, SD, SEM, minimum and maximum values, and 5th–95th percentiles, are provided in the Source Data files.

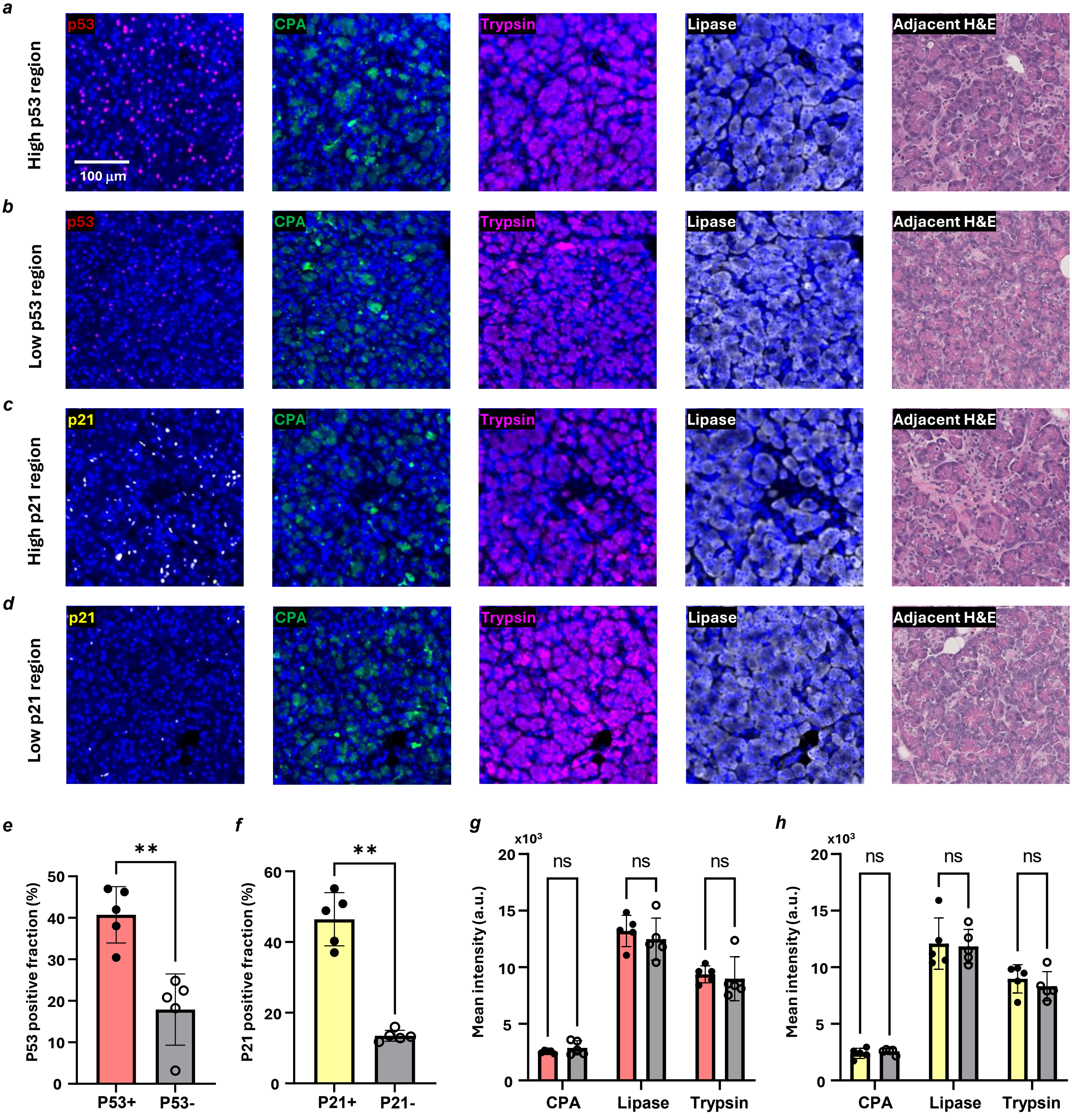

a
b
c
d
e
f
g
h
High p53 region
Low p53 region
High p21 region
Low p21 region
p53
p21
CPA
Trypsin
Lipase
Adjacent H&E
100 µm
P53 positive fraction (%)
P53+
P53-
P21 positive fraction (%)
P21+
P21-
**
ns
Mean intensity (a.u.)
x10³
CPA
Lipase
Trypsin

Supplementary Figure 15: Lack of association between P21+/P53+ cell density and acinar enzyme expression. a-d. Representative iCLAP-CyCIF images co-detecting P53 or P21 with acinar enzyme markers CPA, Trypsin, and Lipase in the P53-high (**a**), P53-low (**b**), P21-high (**c**), and P21-low (**d**) acinar regions. Adjacent H&E staining provides tissue morphology for reference. No visual co-variation between P53/P21 occurrence and enzyme signal was observed. **e-f.** Quantification of P53+ (**e**) and P21+ (**f**) cell density confirms that the designated high vs low regions differ as intended. **g-h.** Mean enzyme intensities across five ROIs per group show no statistically significant differences between P53-high vs P53-low (**g**) or P21-high vs P21-low (**h**) regions. Statistical tests and *P* values are described in the Statistical Analysis section. Two-sided unpaired t-test (Welch's) to evaluate differences in ROI-level P53+ and P21+ occurrence between P53+/- and P21+/- ROIs (Supp. Fig. 15e-f). Two-way analysis of variance (ANOVA) to evaluate the effects of P53/P21 status (P53/P21+ vs P53/P21-) and enzyme marker expression level (CPA, Lipase, and Trypsin) (Supp. Fig. 15g-h). Tukey's multiple comparisons test was applied post hoc to evaluate group differences. Data are presented as mean ± standard deviation (SD) from three biological replicates. Results were considered significant at $P < 0.05$ (*), $P < 0.01$ (**), and $P < 0.001$ (***), $P < 0.0001$ (****). "ns" indicates no significant difference.

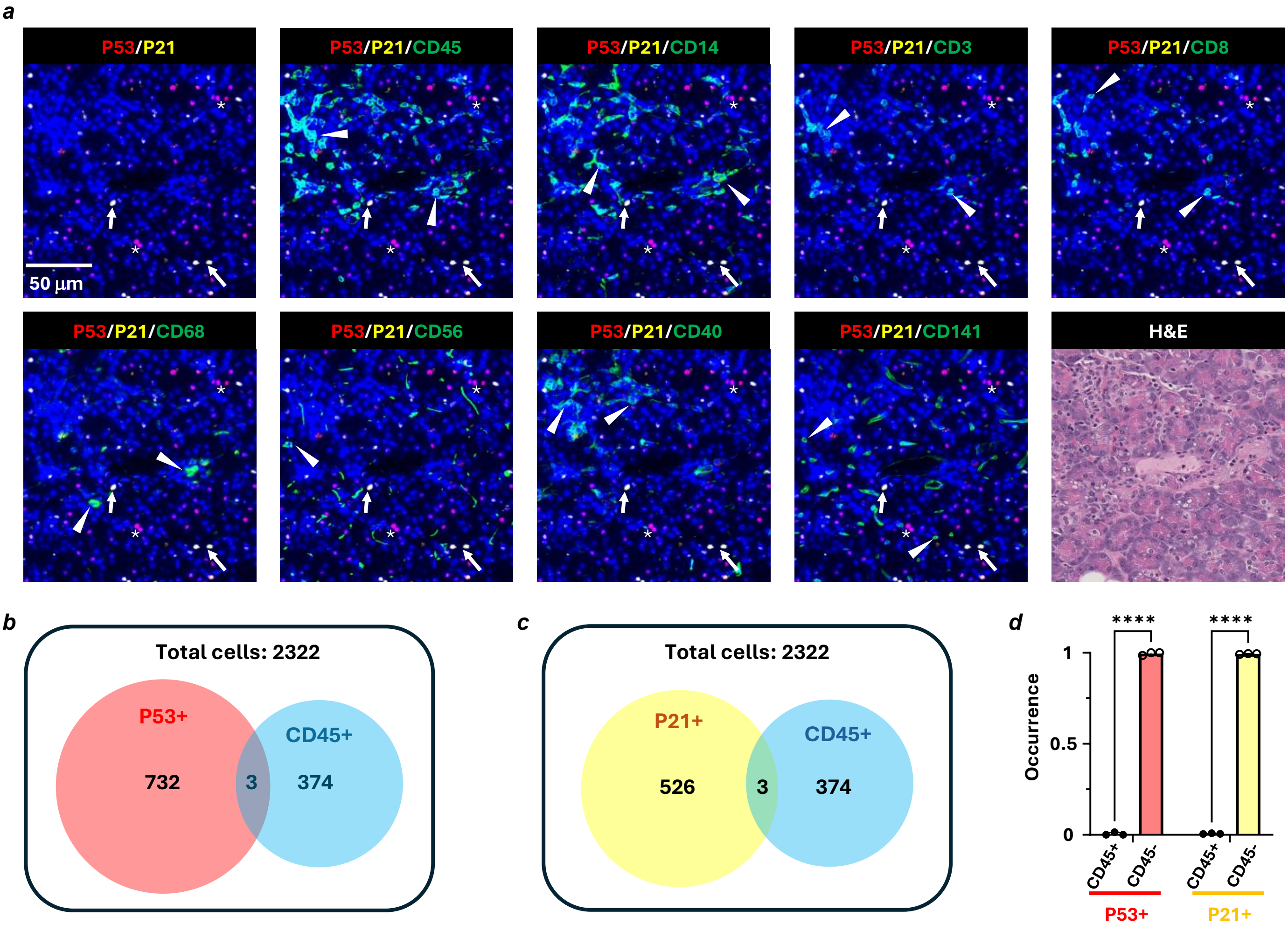

Supplementary Figure 16. P21+ and P53+ acinar cells are distinct from immune cell populations. a. Representative multiplex iCLAP-IF images of human pancreatic tissue sections stained for P21 (yellow) and P53 (red) with immune markers CD3, CD8, CD14, CD40, CD45, CD56, CD68, and CD141 (green) in the acinar region. Arrows indicate P21+ cells, asterisks indicate P53+ cells, and arrowheads indicate immune marker–positive cells. Lack of immune marker expression confirms that P21+ and P53+ cells are non-immune. Adjacent H&E staining is shown for morphology reference. **b-c.** Venn diagrams show minimal overlap between P53+ or P21+ populations and CD45+ immune cells in 3 immune-enriched ROIs (n = 2322 total cells). **d.** Quantification from 3 immune-enriched ROIs (dots = per ROI values; bars = mean ± SD) confirms that P53+ and P21+ cells are enriched in CD45- populations. Two-way analysis of variance (ANOVA) to evaluate the effects of CD45 status (CD45+ vs CD45-) and marker type (P53+ vs P21+) on the proportion of positive cells (Supp. Fig. 16d). Tukey's multiple comparisons test was applied post hoc to evaluate group differences. Data are presented as mean ± standard deviation (SD) from three biological replicates. Results were considered significant at $P < 0.05$ (*), $P < 0.01$ (**), and $P < 0.001$ (***), $P < 0.0001$ (****). "ns" indicates no significant difference.

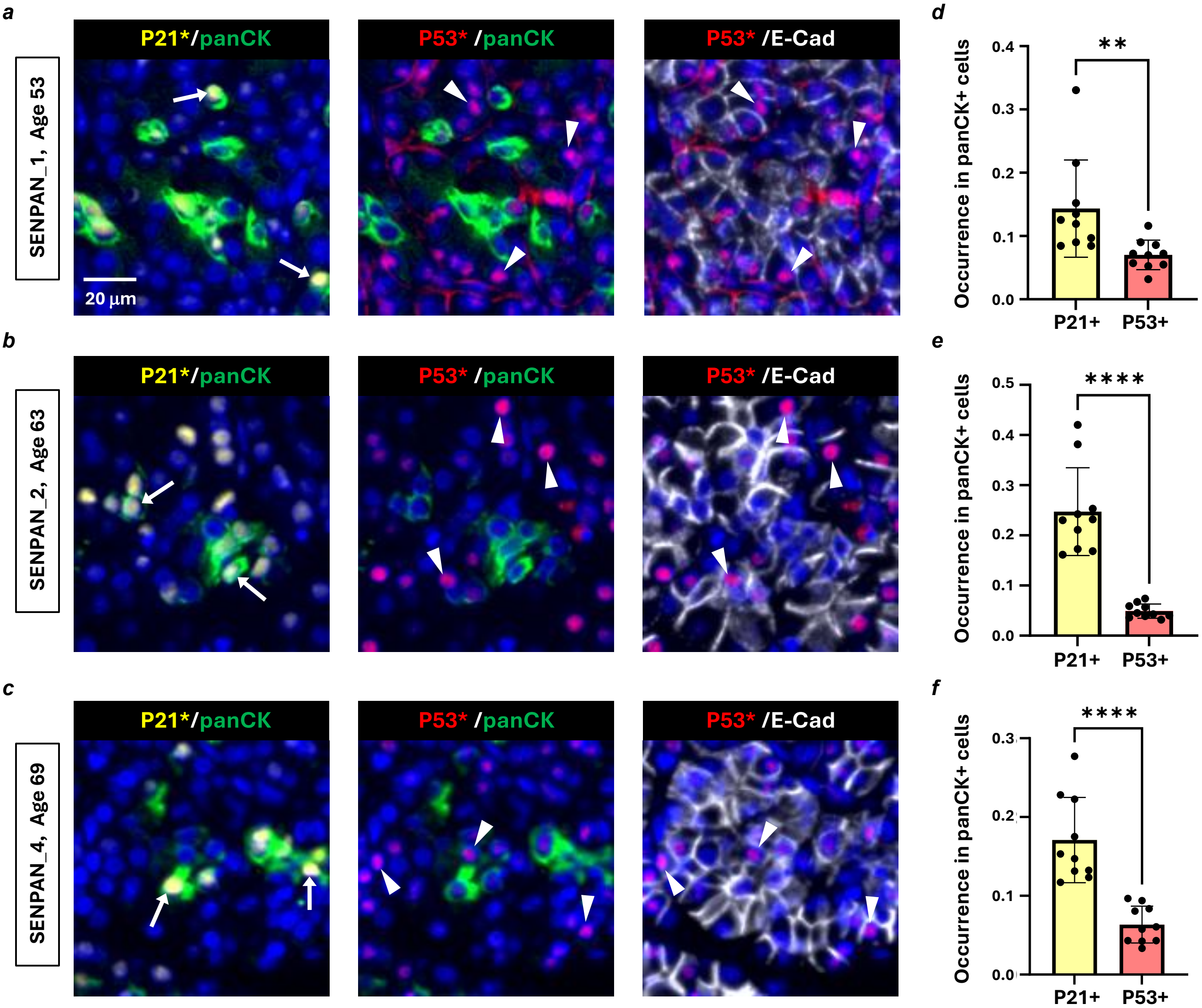

Supplementary Figure 17. Higher P21+ than P53+ occurrence in panCK+ cells across multiple donors. a-c. Representative iCLAP images from three older donors. **d-f.** Quantification (dots = per ROI values; bars = mean ± SD) from 10 non-overlapping ROIs (2000 × 2000 px; ~1.3 × 1.3 mm each) per donor. Within the panCK+ cells, P21+ occurrence was significantly higher than P53+ in all three donors. Statistical tests and *P* values are described in the Statistical Analysis section. Two-sided unpaired t-test (Welch's) to assess P21+ and P53+ occurrence within panCK+ cells in 3 donors. For each donor, we selected and annotated non-overlapping acinar region in 10 acinar ROIs from adjacent H&E-matched fields. Data are presented as mean ± standard deviation (SD) from three biological replicates. Results were considered significant at $P < 0.05$ (*), $P < 0.01$ (**), and $P < 0.001$ (***), $P < 0.0001$ (****). "ns" indicates no significant difference.

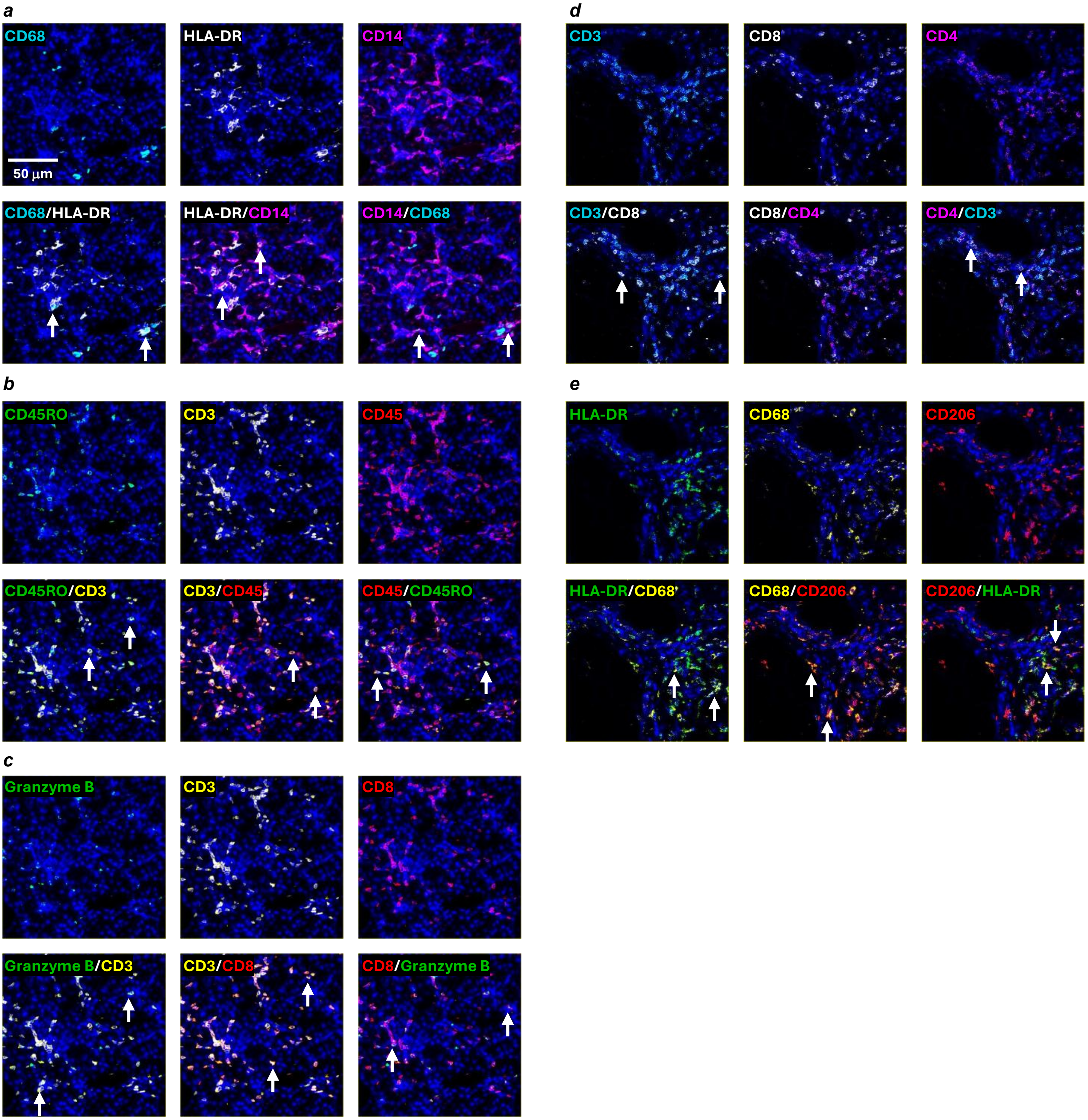

Supplementary Figure 18: Representative CODEX and IMC images staining validation. a. Colocalized CODEX staining of macrophage markers CD68, HLA-DR, and CD14. **b.** Colocalized CODEX staining of T cell markers CD3, CD45, and CD45RO. **c.** Cytotoxic T cell staining using Granzyme B, CD3, and CD8. **d.** IMC validation of T cell subsets with CD3, CD8, and CD4. **e.** IMC staining of macrophage markers HLA-DR, CD68, CD206. Arrows indicate colocalization of markers within specific immune cell populations. Representative images and intensity distributions are shown from at least two independent ROIs with similar pattern.

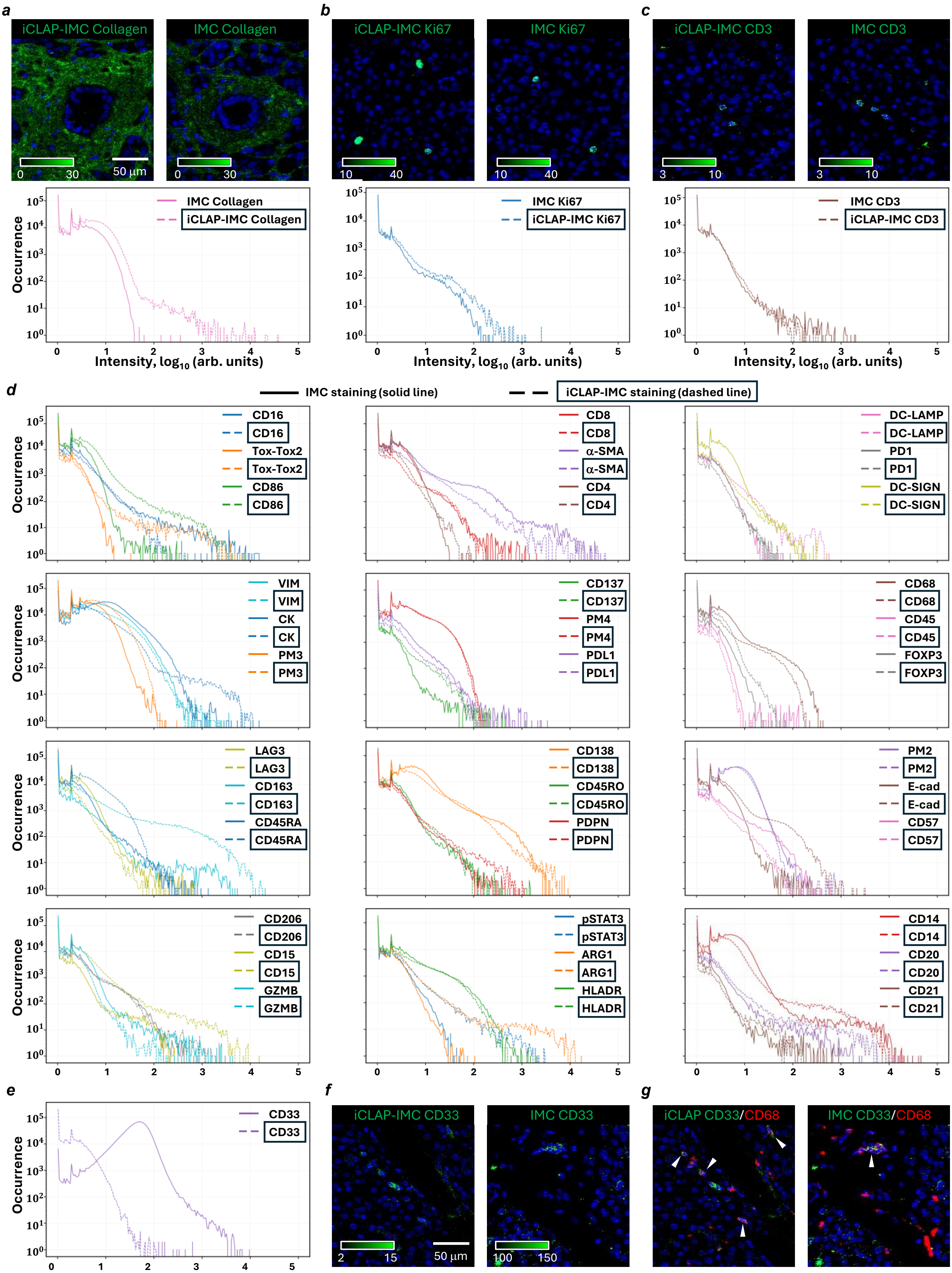
a
iCLAP-IMC Collagen
IMC Collagen
0 30
50 µm
IMC Collagen
iCLAP-IMC Collagen
Occurrence
Intensity, log10 (arb. units)
b
iCLAP-IMC Ki67
IMC Ki67
10 40
IMC Ki67
iCLAP-IMC Ki67
c
iCLAP-IMC CD3
IMC CD3
3 10
IMC CD3
iCLAP-IMC CD3
d
IMC staining (solid line)
iCLAP-IMC staining (dashed line)
CD16
Tox-Tox2
CD86
CD8
α-SMA
CD4
DC-LAMP
PD1
DC-SIGN
VIM
CK
PM3
CD137
PM4
PDL1
CD68
CD45
FOXP3
LAG3
CD163
CD45RA
CD138
CD45RO
PDPN
PM2
E-cad
CD57
CD206
CD15
GZMB
pSTAT3
ARG1
HLADR
CD14
CD20
CD21
e
CD33
f
iCLAP-IMC CD33
IMC CD33
2 15
50 µm
100 150
g
iCLAP CD33/CD68
IMC CD33/CD68

Supplementary Figure 19. Comparison of iCLAP – IMC and conventional IMC staining across a 40 plex IMC panel. a-c. Representative images and signal intensity distributions for Collagen (a), Ki67 (b), and CD3 (c) comparing conventional IMC (solid lines) and iCLAP-IMC (dashed lines). iCLAP-IMC preserves comparable signal intensity with conventional IMC across distinct cellular and extracellular compartments, including the extracellular matrix (Collagen), nucleus (Ki67), and cell membrane (CD3). The pseudocolor intensity scale bar shown at the bottom left indicates fluorescence intensity values (arbitrary units). **d.** Signal intensity distribution plots for 36 additional markers spanning immune, stromal, and epithelial compartments. Across these markers, iCLAP-IMC demonstrates comparable signal intensity and distribution to conventional IMC, indicating effective preservation of marker signal across a broad multiplex panel. **e.** CD33 intensity distribution showing a reduced signal in iCLAP-IMC compared to conventional IMC. **f–g.** Despite the decreased CD33 intensity, colocalization with CD68-positive macrophages is retained, supporting the specificity and interpretability of CD33 staining in iCLAP-IMC.
