# Supplementary material for: iCLAP: an innovative method for integrable co-detection of low-abundance antigens with high-plex immunostaining
Source: Nat Commun. 2026 Feb 24;17:3104. doi: 10.1038/s41467-026-69752-y (PMC13039418; doi:10.1038/s41467-026-69752-y)
Supplement: Supplementary file 2 — Description of additional supplementary files [file 41467_2026_69752_MOESM2_ESM.pdf]

DESCRIPTION OF ADDITIONAL SUPPLEMENTARY FILES

Supplementary Data 1. Human specimen metadata and figure-panel mapping. For each main and supplementary figure panel, the table lists the tissue sample identifier, donor age range and sex, section/slide number, and source—Johns Hopkins Medical Institutions (JHMI), Cellular Senescence Network (SenNet), or AMS Biotechnology Europe Ltd (AMSBIO). Sample prefixes indicate tissue sets (e.g., SENPAN_ = pancreas; SENBRE_ = breast). All specimens are FFPE tissues.

Supplementary Data 2. List of antibodies used in the iCLAP mutiplex immunolabeling, detailing target proteins, vendors, catalog or clone numbers, staining method, working dilution, and detection channel. Targets were stained using either tyramide signal amplification (TSA), conventional immunofluorescence (IF), CODEX barcoding, or Imaging Mass Cytometry (IMC). Detection channels include a range of fluorophores (e.g., CY3, CY5, Alexa Fluor, Atto dyes) or metal isotopes for IMC. The panel covers a wide range of biological targets, including transcription factors, signaling molecules, senescence markers, inflammatory cytokines, and pancreatic cell identity markers.
