# Supplementary material for: iCLAP: an innovative method for integrable co-detection of low-abundance antigens with high-plex immunostaining
Source: Nat Commun. 2026 Feb 24;17:3104. doi: 10.1038/s41467-026-69752-y (PMC13039418; doi:10.1038/s41467-026-69752-y)
Supplement: Supplementary file 5 — Reporting Summary [file 41467_2026_69752_MOESM5_ESM.pdf]

nature portfolio

Corresponding author(s): PeiHsun Wu, Denis Wirtz

Last updated by author(s): May 2, 2025

Reporting Summary

Nature Portfolio wishes to improve the reproducibility of the work that we publish. This form provides structure for consistency and transparency in reporting. For further information on Nature Portfolio policies, see our Editorial Policies and the Editorial Policy Checklist.

Statistics

For all statistical analyses, confirm that the following items are present in the figure legend, table legend, main text, or Methods section.

n/a	Confirmed	
☐	☒	The exact sample size (*n*) for each experimental group/condition, given as a discrete number and unit of measurement
☐	☒	A statement on whether measurements were taken from distinct samples or whether the same sample was measured repeatedly
☐	☒	The statistical test(s) used AND whether they are one- or two-sided<br>*Only common tests should be described solely by name; describe more complex techniques in the Methods section.*
☒	☐	A description of all covariates tested
☒	☐	A description of any assumptions or corrections, such as tests of normality and adjustment for multiple comparisons
☐	☒	A full description of the statistical parameters including central tendency (e.g. means) or other basic estimates (e.g. regression coefficient) AND variation (e.g. standard deviation) or associated estimates of uncertainty (e.g. confidence intervals)
☐	☒	For null hypothesis testing, the test statistic (e.g. *F*, *t*, *r*) with confidence intervals, effect sizes, degrees of freedom and *P* value noted<br>*Give P values as exact values whenever suitable.*
☒	☐	For Bayesian analysis, information on the choice of priors and Markov chain Monte Carlo settings
☒	☐	For hierarchical and complex designs, identification of the appropriate level for tests and full reporting of outcomes
☐	☒	Estimates of effect sizes (e.g. Cohen's *d*, Pearson's *r*), indicating how they were calculated

Our web collection on statistics for biologists contains articles on many of the points above.

Software and code

Policy information about availability of computer code

Data collection	Fluorescent Image acquisition was performed with NIS-element software (Nikon);<br>A Lumencor SpectraX 6<br>S Fluor 10× objective (NA 0.5; MRF00100, Nikon)<br>The Perfect Focus System (Nikon)
Data analysis	Previously established custom MATLAB scripts were used for image stitching, image alignment, cell segmentation, and intensity feature extraction extraction. Data analysis code are available in Code Availability section.<br>GraphPad Prism 9<br>NDP software for FFPE HE staining<br>QuPath

For manuscripts utilizing custom algorithms or software that are central to the research but not yet described in published literature, software must be made available to editors and reviewers. We strongly encourage code deposition in a community repository (e.g. GitHub). See the Nature Portfolio guidelines for submitting code & software for further information.

Data

Policy information about availability of data

All manuscripts must include a data availability statement. This statement should provide the following information, where applicable:

- Accession codes, unique identifiers, or web links for publicly available datasets
- A description of any restrictions on data availability
- For clinical datasets or third party data, please ensure that the statement adheres to our policy

The data will be made available upon request.

Research involving human participants, their data, or biological material

Policy information about studies with human participants or human data. See also policy information about sex, gender (identity/presentation), and sexual orientation and race, ethnicity and racism.

Reporting on sex and gender	The sex, age, and gender information were provided in Supplementary Data 1.
Reporting on race, ethnicity, or other socially relevant groupings	N/A
Population characteristics	See Supplementary Data 1.
Recruitment	Human whole pancreas from anonymized donors were obtained thruough the University of Texas Health San Antonio or Imagine Pharma.
Ethics oversight	This study was approved by the Johns Hopkins School of Medicine Institutional Review Board. For whole pancreas analysis the organ is collected from UT Health San Antonio (UTHSCSA), the Center for Life Donor Biorepository was determined to be Non-Human Research by the UTHSA IRB, as the donors are deceased at time of inclusion. The pancreata were obtained through this biobank protocol at University Hospital Center for Life, which is managed in partnership between University Health Transplant Institute (UHTI) and Texas Organ Sharing Alliance (TOSA), who is the local Organ Procurement Organization. TOSA has jurisdiction over the donors, and an approval was received from their Advisory Board to receive organs intended for research.

Note that full information on the approval of the study protocol must also be provided in the manuscript.

Field-specific reporting

Please select the one below that is the best fit for your research. If you are not sure, read the appropriate sections before making your selection.

☒ Life sciences ☐ Behavioural & social sciences ☐ Ecological, evolutionary & environmental sciences

For a reference copy of the document with all sections, see nature.com/documents/nr-reporting-summary-flat.pdf

Life sciences study design

All studies must disclose on these points even when the disclosure is negative.

Sample size	Five pancreas tissue specimen and six TMA samples were used in this work. Two pancreas sample were selected from archival tissue Johns Hopkins School of Medicine, and another three samples were collected by Harvard University. The TMA samples were purchased from TissueArry.com. One breast and one pancreas sample used for tissue integrity testing were purchased from AMSbio.
Data exclusions	No data was excluded from this work.
Replication	The main multiplex staining results were replicated in multiple sections and also validated with other methods. Sample size and number of experimental repeats were indicated in the figure legends. iCLAP staining performance, signal amplification, fluorophore inactivation, and multiplex compatibility were validated on independent FFPE tissue sections from multiple donors and tissue types, with consistent staining patterns and quantitative trends observed. Representative imaging results shown in the figures were reproduced in at least three independent tissue samples unless otherwise stated. Quantitative analyses were performed using standardized image processing pipelines and identical analysis parameters across datasets. All attempts at replication were successful and no irreproducible findings were identified.
Randomization	Randomization is not applicable in this study. This study analyzed archived human tissue specimens without experimental manipulation or intervention. Grouping was based on biological characteristics measured directly from the data (e.g., marker expression status, cell type, or tissue region). Regions of interest were selected based on tissue quality and presence of relevant structures. Because no treatment assignment or intervention was performed, randomization and allocation concealment were not applicable.
Blinding	No blinding is used. Samples were not assigned to experimental interventions and analyses were algorithm-driven, blinding was not considered necessary or applicable.

Reporting for specific materials, systems and methods

We require information from authors about some types of materials, experimental systems and methods used in many studies. Here, indicate whether each material, system or method listed is relevant to your study. If you are not sure if a list item applies to your research, read the appropriate section before selecting a response.

Materials & experimental systems

n/a	Involved in the study
☐	☒ Antibodies
☒	☐ Eukaryotic cell lines
☒	☐ Palaeontology and archaeology
☒	☐ Animals and other organisms
☒	☐ Clinical data
☒	☐ Dual use research of concern
☒	☐ Plants

Methods

n/a	Involved in the study
☒	☐ ChIP-seq
☒	☐ Flow cytometry
☒	☐ MRI-based neuroimaging

Antibodies

Antibodies used

All antibodies used are listed in supplemental table 2.

Validation

TSA staining results were validated using immunohistochemistry performed on the same samples by the Oncology Tissue Services Core Lab at Johns Hopkins University (Supplementary Figure 3). The iCLAP-CyCIF results were validated through assessment of spatial distribution consistency with know tissue architecture from adjacent H&E images (Figure 4). The iCLAP-CODEX and iCLAP-IMC results were validated through colocalization analysis of canonical marker pairs and assessment of spatial distribution consistency with known tissue architecture (Supplementary Figure 18).

Plants

Seed stocks

NA

Novel plant genotypes

NA

Authentication

NA
